# Molecular Basis of Impaired Glycogen Metabolism during Ischemic Stroke and Hypoxia

**DOI:** 10.1371/journal.pone.0097570

**Published:** 2014-05-23

**Authors:** Mohammed Iqbal Hossain, Carli Lorraine Roulston, David Ian Stapleton

**Affiliations:** 1 Department of Physiology, St. Vincent's Campus, The University of Melbourne, Melbourne, Victoria, Australia; 2 Department of Medicine, St. Vincent's Campus, The University of Melbourne, Melbourne, Victoria, Australia; Glasgow University, United Kingdom

## Abstract

**Background:**

Ischemic stroke is the combinatorial effect of many pathological processes including the loss of energy supplies, excessive intracellular calcium accumulation, oxidative stress, and inflammatory responses. The brain's ability to maintain energy demand through this process involves metabolism of glycogen, which is critical for release of stored glucose. However, regulation of glycogen metabolism in ischemic stroke remains unknown. In the present study, we investigate the role and regulation of glycogen metabolizing enzymes and their effects on the fate of glycogen during ischemic stroke.

**Results:**

Ischemic stroke was induced in rats by peri-vascular application of the vasoconstrictor endothelin-1 and forebrains were collected at 1, 3, 6 and 24 hours post-stroke. Glycogen levels and the expression and activity of enzymes involved in glycogen metabolism were analyzed. We found elevated glycogen levels in the ipsilateral hemispheres compared with contralateral hemispheres at 6 and 24 hours (25% and 39% increase respectively; *P*<0.05). Glycogen synthase activity and glycogen branching enzyme expression were found to be similar between the ipsilateral, contralateral, and sham control hemispheres. In contrast, the rate-limiting enzyme for glycogen breakdown, glycogen phosphorylase, had 58% lower activity (P<0.01) in the ipsilateral hemisphere (24 hours post-stroke), which corresponded with a 48% reduction in cAMP-dependent protein kinase A (PKA) activity (P<0.01). In addition, glycogen debranching enzyme expression 24 hours post-stroke was 77% (P<0.01) and 72% lower (P<0.01) at the protein and mRNA level, respectively. In cultured rat primary cerebellar astrocytes, hypoxia and inhibition of PKA activity significantly reduced glycogen phosphorylase activity and increased glycogen accumulation but did not alter glycogen synthase activity. Furthermore, elevated glycogen levels provided metabolic support to astrocytes during hypoxia.

**Conclusion:**

Our study has identified that glycogen breakdown is impaired during ischemic stroke, the molecular basis of which includes reduced glycogen debranching enzyme expression level together with reduced glycogen phosphorylase and PKA activity.

## Introduction

Stroke is the second most common cause of death and disability world-wide making it the most complex and devastating of all neurological diseases [1], [2]. In spite of decades of intensive research in developing pharmacological interventions that prevent damage due to stroke, very few therapeutic options are currently available. Our lack of knowledge about cellular energy utilization during the excitotoxic phase of stroke is one of the many reasons for the lack of therapeutic success. The primary effect of ischemic stroke to neurons is a massive shortage of energy supply with rapid depletion of glucose and oxygen, which impairs the synthesis of ATP through glycolysis and oxidative phosphorylation [3].

The severity of the pathological consequences in stroke depends on the availability of alternative energy substrates and the rate of consumption of ATP by cells undergoing ischemic insults. Glycogen is the principal endogenous source of cellular energy alternative to blood-derived glucose during periods of energy-deficiency in the brain [4]. It is a complex branched polymer of glucose found predominantly in the liver and skeletal muscle where it maintains blood glucose homeostasis and provides energy for muscle contractions, respectively. It consists of glucose molecules covalently linked to α-1,4-glycosidic linkages attached to the polypeptide chain that are extended by the collaborative actions of glycogen synthase (GS) and glycogen branching enzyme (GBE) [5], [6]. Glycogen degradation is mediated by the concerted action of glycogen phosphorylase (GP) and glycogen debranching enzyme (GDE) [5], [6].

Brain glycogen storage plays a significant role in both physiological and pathological circumstances such as wakefulness [7], hypoglycemia [8], cerebral ischemia [4] and learning [9] by providing energy to adjacent neurons and axons [10], [11]. It also provides protection against reactive oxygen species produced during cellular injury [12]. Studies using cell culture and optic nerve preparations have demonstrated that elevated glycogen improves neuronal survival and axonal function during glucose deprivation [13]. Glycogenolysis also supports the *de novo* synthesis of glutamate in astrocytes during glutamatergic activity [14]. Indeed glycogen-derived carbons are used for anaplerosis via pyruvate carboxyaltion in order to produce glutamate [14], [15].

According to the Astrocyte-Neuron Lactate Shuttle Hypothesis (ANLSH), lactate or pyruvate derived from astrocytic glycogen upon stimulation of neurons by neurotransmitter glutamate, can be shuttled to neurons for oxidative metabolism to provide energy for maintaining ionic gradients and synaptic plasticity [4], [13]. In contrast, based on the concentration and kinetic characteristics of the blood-brain barrier, neuronal and glial nutrient transporter proteins that specifically mediate brain glucose and lactate transport, Simpson et al. (2007) proposed that neurons metabolize glucose and export lactate to astrocytes [16]. This hypothesis is further supported by Mangia et al. (2009) who demonstrated shuttling of lactate from neurons to astrocytes (NALS) in the human brain during visual stimulation using proton Magnetic Resonance Spectroscopy (^1^H MRS) [17]. An elegant mathematical modelling by DiNuzzo et al. (2010) suggests that glycogen can be used as a significant source of energy for astrocytes and can act as a regulator of astrocytic utilization of blood-born glucose [18], [19]. According to the model, glycogenolysis regulates utilization of extracellular and blood-born glucose by astrocytes during brain stimulation by inhibition of astrocytic hexokinase and makes extracellular and blood-born glucose available for use by neurons to meet the immediate demand of energy during brain stimulation [18], [20]. Glycogen in astrocytes also plays a significant role in the sequestration of excess extracellular K^+^ released by neurons during action and synaptic potentials [21]. It is also used as a source of energy by astrocytes to scavenge excess glutamate during ischemic stroke-induced excitotoxicity [10]. For these reasons previous studies have reported that glycogen levels and metabolism is affected in pathological conditions such as cancer (gastric adenocarcinoma) and neurological disease (Alzheimer's disease and AIDS dementia) [22]–[25].

Little is known about the fate of glycogen and regulation of its metabolizing enzymes during ischemic stroke. Here, we report changes in the glycogen level and its associated metabolizing enzymes during stroke with reperfusion and investigate the signaling pathways involved in this energy-deficient pathology in the search for better treatments post-stroke.

## Materials and Methods

### Animals

All experiments involving animals were approved by the St. Vincent's Hospital Animal ethics committee, The University of Melbourne and were performed in accordance with the Prevention of Cruelty to Animals Act 1986 under the guidelines of the National Health and Medical Research Council Code of Practice for the Care and Use of Animals for Experimental Purposes in Australia. Male hooded Wistar rats were purchased from the University of Adelaide, Laboratory Animal Services, Australia. Rats were group-housed (4 rats to a cage) in the Biological Research Facility at The University of Melbourne until endothelin-1-induced (ET-1) middle cerebral artery constriction. Following stroke, they were housed in separate cages under diurnal lighting with ambient temperature maintained between 20 and 22°C and given free access to food and water.

### Surgical implantation of ET-1 guide cannula

Male hooded Wistar rats, aged 10–12 weeks (n = 5/6 per groups, total 35/40) (300–340 g) were anesthetized with Ketamine/Xylazine (75 mg/kg Ketamine/10 mg/kg Xylazine *i.p.*) and maintained throughout surgery by inhalation isoflurane (95% oxygen and 2% isoflurane). A 23-gauge stainless-steel guide cannula was stereotaxically implanted into the piriform cortex 2 mm dorsal to the right middle cerebral artery (0.2 mm anterior, −5.2 mm lateral and −5.9 mm ventral) as described previously [26]–[28] for later delivery of ET-1 in conscious rats. Control rats underwent cannula implantation and saline infusion instead of ET-1 also described previously [26], [27]. All surgery was performed under general anesthesia and all efforts were made to minimize suffering which included access to paracetomol (2 mg/kg in drinking water) 24 hours prior to and after surgery, as well as extensive monitoring of each rat throughout the duration of the study.

### Stroke induction by ET-1 in conscious rats

Focal cerebral ischemia was induced in the conscious rats by constriction of the right middle cerebral artery with perivascular administration of ET-1 (60 pmol in 3 µl of saline over 10 min). Rats were placed in a clear Plexiglass box for observation during ET-1 injection. During stroke induction, counter-clockwise and/or clockwise circling, clenching, and dragging of the contralateral forepaw were observed, validating the correct placement of the cannula. Stroke severity was scored based on these responses, which have been shown to be a reliable prediction of stroke outcome [26]. Rats that did not display contralateral circling indicative of moderate to severe stroke were excluded from the study. Rectal temperatures were taken with a thermistor probe, prior to stroke and at 30 minutes intervals for 3 to 5 hours after stroke and maintained around 37°C (Figure S1 in Supporting Information S1) as described in previous studies [26], [27]. Body weight was also recorded pre-surgery, pre-stroke and 24 hours post-stroke where appropriate.

### Tissue collection

Rats were decapitated at 1, 3, 6 and 24 hours after stroke induction and forebrains were removed. Sham rats were also decapitated at these time points as control groups. The left and right hemispheres were separated and immediately snapped freeze in liquid nitrogen. Frozen tissues were powdered under liquid N_2_ in a mortar kept on dry ice using a pestle precooled in liquid N_2_ and proteins were extracted by homogenization with the ice-cold lysis buffer [50 mM Tris (pH 7.0), 1 mM EDTA, 5 mM EGTA, 1 mM dithiothreitol, 10% Glycerol, 0.1% Triton X-100, 150 mM NaCl, 50 mM NaF, 40 mM sodium pyrophosphate, 0.5 mM Na_3_VO_4_, 50 mM β-glycerophosphate, 0.2 mg/ml benzamidine, 0.1 mg/ml phenymethylsufonyl fluoride (PMSF), EDTA-free protease inhibitors and phosphatase inhibitors cocktail (Roche, Indianapolis, IN, USA)]. After centrifugation at 10,000×g for 10 minutes, supernatant was collected and total protein concentration was determined using Bradford assay (Bio-Rad, Hercules, CA, USA). The supernatant was stored at −20°C.

### Rat primary cerebellar astrocyte culture

Rat primary astrocytes were cultured from the cerebellar hemispheres of 1- to 2-day old Wistar rat brains (n = 6/7 per culture) according to the methods described previously [29], [30]. Briefly, cerebella were rapidly excised and transferred to an ice cold Ca^2+^-free isotonic salt solution [29]. After removing meninges, the cerebella were digested using DMEM (Sigma-Aldrich, USA) containing 0.1% trypsin for 5 minutes at room temperature in a sterile dish. Trypsin digestion was stopped by adding Eagle's Basal Medium (BME) supplemented with 10% fetal calf serum (FCS) (Sigma-Aldrich, USA). The tissue was then dissociated by triturating using successively decreasing bore sized fire-polished Pasteur pipettes. The cells were centrifuged at 600 rpm for 5 minutes and resuspended in DNAse containing solution. Centrifugation was repeated and the pellet was resuspended in DMEM (supplemented with 2.0 g of NaHCO_3_, 2.0 g of glucose, 1000 U/L penicillin, 1 g/L streptomycin, and 5% FCS). The cells were plated in poly-D-lysine (PDL)-coated (0.001%) flasks (Invitrogen, USA) with DMEM and incubated at 37°C in a humidified atmosphere with 5% CO_2_. The cells were maintained there for 7–8 days to allow for growth and proliferation to 60–70% confluency. The medium was changed at every three days until the cells were confluent at the desired level. The confluent cells were shaken overnight at 360 rpm at 37°C and washed with sterile PBS three times to remove oligodentrocytes. Fresh culture medium (DMEM supplemented with 10% FCS and 6 mM glucose) was then added to the pure astrocytes and the cultures were incubated for 24 hours. The next day astrocyte cultures were trypsinized and re-plated on to poly-D-lysine coated plates at a density of 2×10^4^ cells per cm^2^. With this method, the cells in the culture contained ≥95% astrocytes as revealed by immunocytochemistry using anti-GFAP antibody (1∶300) (Figure S2 in Supporting Information S1). The medium was changed at every three days and all the experiments with astrocytes were performed at DIV 19–22.

### Rat primary cortical neuronal culture

Rat primary cortical neuronal culture was performed based on the previously described method with modification [27]. Pregnant rats (n = 3) (gestational day 16–18) were euthanized by CO_2_ aphyxiation and the embryos (n = 7/8 per rat) were collected by Cesarean section. Briefly, the cortical regions of the embryonic brains were aseptically dissected, freed of meninges and dissociated in Ca^2+^-free Suspension Buffer [250 ml of Hanks balanced salt solution (HBSS) +1.94 ml of 150 mM MgSO_4_+0.75 g BSA], subjected to trypsin digestion at 37°C for 5 minutes in Trypsin Digestion Buffer [20 ml Suspension Buffer +80 µl DNAse (10 µg/µl), +4 mg trypsin]. Tryptic digestion was stopped by the addition of Trypsin Inhibitor Buffer (16.8 ml of Suspension Buffer +80 µl DNAse, +200 µl of 150 mM MgSO_4_+10.4 mg trypsin inhibitor) to the cell suspension and centrifuged at 1000×g for 5 minutes at 23°C. The pelleted sample was then subjected to mechanical trituration in Trypsin Inhibitor Buffer for 30 seconds. The cells were collected by centrifugation at 1000×g for 5 minutes at 23°C. Finally, the cells were re-suspended in pre-warmed (37°C) Neurobasal medium (GIBCO, USA) supplemented with 10% FCS, 0.25% GlutaMAX-I (GIBCO, USA) and 1% penicillin and streptomycin. The cells were plated to a density of 5×10^7^cells in a petri dish (57 cm^2^) (Nunc) previously coated with 0.1 mg/ml sterile poly-D-lysine. The cultures were maintained at 37°C in 5% CO_2_ and 95% air in a humidified incubator. After 24 hours, the initial Neurobasal medium was replaced with the medium containing 2.5% B-27, (Invitrogen) 0.25% GlutaMAX-I and 1% penicillin and streptomycin. This medium supports long-term survival (several weeks) of neurons and suppresses the growth (< 2%) of glial cells in the total population. The old medium was replaced with new medium at every 4 days.

### Induction of hypoxia

*In vitro* hypoxia/ischemia was induced by placing the cerebellar astrocyte cultures into a modular hypoxic incubator containing 2% O_2_, 5% CO_2_ and 93% N_2_ gas mixture for 0 (control), 1, 3 6 and 24 hours. No decrease in pH below 7.2 was observed during hypoxia. Re-oxygenation was induced by returning the cultures to the normoxia incubator for 24 hours. The culture medium was replaced with glucose- and serum-free DMEM prior to hypoxia or normoxia.

### PKA inhibitor treatment

Stock solutions (10x) of a cell permeable, potent and specific PKA inhibitors, H89 (*N*-[2-(*p*-bromo-cinnamylamino)-ethyl]-5-isoquinolinesulfonamide dihydrochloride) (Sigma-Aldrich, USA) and Rp-8-Cl-cAMPs (8-Chloroadenosine-3′, 5′-cyclic monophosphorothioate, Rp-isomer) (Calbiochem, USA) were prepared by dissolving in DMSO and milliQ water, respectively. Astrocytes were incubated in the culture medium (DMEM supplemented with 10% FCS and 6 mM glucose) containing H89 (1, 2, and 4 µM) or Rp-8-Cl-cAMPs (50, 100 and 150 µM) for 24 hours to monitor the effect of PKA inhibition.

### Glycogen quantification

Total glycogen concentration was determined by colorimetric method based on the free glucose present in the lysates as previously described [23]. Briefly, 100 µl of brain/cell lysate was treated with 5 µl α-amyloglucosidase (10 µg/µl) in buffer containing 5 µl sodium acetate (pH 6.0). Samples without α-amyloglucosidase were used as negative controls. Both types of samples were incubated at 50°C for 20 minutes. After incubation samples were centrifuged at 14,000×g for 2 minutes and 50 µl of the supernatant were transferred to another microcentrifuge tube into which 350 µl PGO (Sigma-Aldrich, USA) reaction buffer containing O-dianisidine dihydrochloride was added. The mixture was further incubated for 20 minutes at 50°C to develop color. Standard curve was prepared using known concentration of glucose (5.56 mmol/l). Absorbance was taken at 450 nm using xMark micorplate absorbance spectrophotometer (Bio-Rad Inc. USA). Glucose content was calculated by plotting the absorbance values of the samples into standard curve and fitted with linear equation. Glycogen concentration was determined by subtracting values of negative assays (without α-amyloglucosidas), representing bound glucose to positive assays that represents free glucose. Glycogen concentration was expressed as µg glycogen per mg of protein in each sample.

### Glycogen synthase (GS) activity assay

GS enzyme activity was determined by measuring the incorporation of [^14^C]-glucose from (UDP-[^14^C]-glucose) into glycogen [31]. The reaction was carried out in the absence or presence of the allosteric activator, glucose-6-phosphate (G6P). The enzyme (GS) is active (dephosphorylated form) when G6P is present and inactive when G6P is not present in the reaction. The ratio of (GS activity – G6P)/(GS activity + G6P), therefore provides the proportion of GS activation in the sample. To measure GS activity, 20 µl of brain/cell lysate was added to 40 µl of reaction buffer [50 mM Tris-HCl (pH 7.8), 25 mM potassium fluoride, 20 mM EDTA, 10 mg/ml glycogen, 6.7 mM UDP-[^14^C]- glucose] either with or without 10.8 mM glucose-6-phosphate. The reaction is allowed to proceed for 30 minutes at 30°C and was terminated by precipitation of glycogen with cold (−20°C) ethanol (66%) and 50 µl of the reaction mix were put on small squares (2.5 cm×2.5 cm, approximately) of filter papers (Whatman 3031–915) and quickly put into cold ethanol (66%). The papers were washed three times in ethanol, one time with acetone and air dried. The reading was performed in scintillation liquid. GS activity was expressed as the ratio of (GS activity – G6P)/(GS activity + G6P), designated as I/T.

### Glycogen phosphorylase (GP) activity assay

GP activity assay was measured fluorometrically using coupled enzyme systems involving phosphoglucomutase and glucose-6-phosphate dehydrogenase that assist reduction of NAD^+^ to NADH [32]. This assay measures enzyme activity in the glycogenolytic direction with the coupled enzyme reactions of phosphoglucomutase and glucose-6-phosphate dehydrogenase. The resulting reduction of NAD^+^ to NADH is then measured. The reaction was started by adding 10 µl of brain/cell lysate into the reaction buffer [50 mM sodium glycerol-phosphate (pH 7.1), 10 mM potassium phosphate, 5 mM MgCl_2_, 0.5 mM NAD^+^, 1 mM DTT, 1.6 unit phosphoglucomutase, 1.6 unit glucose-6-phosphate dehydrogenase, and 0.2% glycogen] in a total volume of 300 µl of reaction mixture. The reaction was monitored by measuring the increase of fluorescence (NADH generation) (excitation 350 nm, emission 470 nm) in a Fluorskan Ascent fluorescence spectrophotometer (Thermo Electron corp.) and fluorescence was measured at every 2 minutes for 1 hour. The results were plotted into a fluorescence (FLU) vs time graph. The slope of the curve representing the reaction velocity was calculated by linear equation and normalized to protein concentration and time.

### Immunoblot

Equal amounts of protein were loaded in each well of 4–15% SDS-PAGE gel and separated using running buffer [25 mM Tris-HCl (pH 7.4), 192 mM glycine, 10% (w/v) SDS] for approximately 60 minutes at 150 V, and then transferred onto PVDF membrane by Turbo Trans blot system (Bio- Rad). The membrane was then blocked with 5% (w/v) non-fat dry milk/BSA in Tris buffered saline with Tween 20 (TBST) [0.2 M Tris-HCl (pH 7.4), 1.5 M NaCl, and 0.1% Tween 20]. After blocking and washing with TBST, the membrane was probed with respective primary antibodies (Table 1) for overnight at 4°C. The membrane was again washed with TBST three times before probing with horseradish peroxidase-conjugated secondary antibodies for 1 hour at 25°C. Protein bands were visualized using chemiluminescence (ECL, Amersham Biosciences) according to the manufacturer's instruction. Images were taken using ChemiDoc MP analyzer using the Image Lab 4.0 software (Bio-Rad).

**Table 1:** Primary and secondary antibodies used in immunoblots.

Antibody	Immunogen	Host	Dilution	Source
Anti-GBE	GBE (189–202) RPKKPRSLRIYES	Rabbit	1∶2000	Stapleton D et al.[60]
Anti-GDE	GDE (59–73) REKFRSLDWENPTE	Rabbit	1∶1000	Stapleton D et al.[60]
Anti-GP	GP (804–818) NIATSGKFSSDRTI	Rabbit	1∶500	Parker GJ et al.[61]
Anti-pGP	GP (9–21) phospho-S^14^ KRKQIS^PO4^VRGL	Rabbit	1∶500	Parker GJ et al.[61]
Anti-p-PKA substrate	Synthetic phospho-PKA substrate peptides	Rabbit	1∶1000	Cell Signaling, USA
Anti-GFAP	Full length human GFAP	Rat	1∶300	Abcam, USA
Anti –β-Actin	N-terminal residues of human β-actin	Mouse	1∶1000	Cell Signaling, USA
Anti-rabbit	Whole IgG-HRP conjugated	Rabbit Fc	1∶5000	GE Healthcare, UK
Anti-mouse	Whole IgG-HRP conjugated	Mouse Fc	1∶5000	GE Healthcare, UK

### PKA activity assay

PKA activity assay was performed using ProFluro PKA assay kit (Promega, USA) according to the manufacturer's instruction (Details have been described in Supporting Information S1).

### Real-time RT-PCR

The mRNA expressions of genes for GBE (*Gbe1*), GS (*Gys1*), GDE (*Agl*) and GP (*Pygb*) were determined by real-time RT-PCR. Total RNA was extracted from 15 to 20 µg of contralateral and ipsilateral hemispheres of sham and stroke-induced rat brains (n = 5 for each group) using a commercially available kit (PureLink RNA Mini Kit, Invitrogen, Carlsbad, CA, USA), according to the manufacturer's instructions. RNA integrity was evaluated on Agilent 2100 Bioanalyzer (Agilent Technologies, Santa Clara, CA) and quantification of RNA was determined using Nanodrop ND-2000 spectrophotometer (Thermo Fisher Scientific). All of the samples showed 260/280 ratio values around 2, which corresponds to pure RNA. RNA Integrity Number (RIN) values were between 9.6 and 10, suggesting RNA samples with high integrity. The RNA samples were stored at −80°C. Complementary DNA (cDNA) was prepared from 1 µg of RNA using the iScript cDNA Synthesis Kit (Bio-Rad Laboratories, Hercules, CA) according to the manufacturer's instructions and was stored at –20°C for subsequent analysis. Real-Time PCR was performed using the Bio-Rad iCycler Thermal Cycler (Bio-Rad Laboratories, Hercules, CA). Primers' sequences are shown in Table 2. The reaction consisted of 4 minutes at 95°C (one cycle) and then 50 cycles of 95°C for 15 sec, 65°C for 15sec, 72°C for 30 sec. PCR was conducted in a reaction mixure containing SYBR Green (iQ SYBR Green Supermix, Bio-Rad), forward and reverse primers (0.5 µM), and cDNA template (5 ng). Measurements included a no-template control as well as an RT (reverse transcription) negative control. For each cDNA sample, three technical replicates were averaged and dissociation curves were routinely performed to check for the presence of a single peak corresponding to the required amplicon. The content of single stranded DNA (ssDNA) in each sample was determined using the Quanti-iT OliGreen ssDNA Assay Kit (Molecular Probes, Eugene, OR, USA), according to the manufacturer's instruction. Gene expression was quantified by normalizing to the cDNA content of each sample and expressed as arbitrary units (AU).

**Table 2:** Primers used in real-time RT-PCR.

Gene	Forward (5-3)	Reverse (5-3)
Glycogen Phosphorylase (*Pygb*)	TCAGGGATGTAGCCAAGGTC	AGCCATGTGGTAACCAGGAG
Glycogen Debranching Enzyme (*Agl*)	CTCACCAGCTGTCATCAGGA	GTGGAGCAGGGACTCTTCTG
Glycogen Branching Enzyme (*Gbe1*)	TTTTCAACTTCCACCCAAGC	CAAAAGCCTCGGCAAAGTAG
Glycogen Synthase (*Gys1*)	CCCCCAATGGACTAAATGTG	AGCGTCCAGCGATAAAGAAA

### Treatment of astrocytes with GP inhibitors

To monitor the effect of glycogen during hypoxia, astrocytes were treated with the GP inhibitors, CP-316819 (5-Chloro-N-[(1S, 2R)-2-hydroxy-3-(methoxymethylamino)-3-oxo-1-(phenylmethyl) propyl]-1H-indole-2-carboxamide) or DAB (1,4-dideoxy-1,4-imino-D-arabinitol hydrochloride) (Sigma-Aldrich, USA) based on previous studies [8], [33]. DMSO (vehicle) and milliQ water were used to make a 10× stock concentrations of CP-316819 and DAB, respectively. The cerebellar astrocytes (DIV 18–19, 2×10^4^ cells per well) were incubated in the culture medium (DMEM supplemented with 10% FCS and 6 mM glucose) containing either DMSO, or CP-316819 (10 µM) or DAB (1 mM) separately for 24 hours. After incubation the astrocyte cultures were washed twice with fresh culture medium. These cultures along with untreated cultures were then incubated in serum- and glucose-free DMEM and placed in the hypoxic incubator for 0 (control), 1, 3, 6 and 24 hours for induction of hypoxia.

### MTT assay

The cells in each well were monitored for viability by the MTT assay. Briefly, MTT reagent was dissolved in RPMI 1640 medium (without phenol red) at a stock concentration of 5 mg/ml and filtered using 0.22 µm to remove insoluble residues as described previously [34]. After induction of hypoxia for different time points, MTT solution equal to 10% (v/v) of the volume of culture medium (0.5 mg/ml final concentration) was added to each well containing astrocytes. The culture plates were then incubated at 37°C in a 5% CO_2_ incubator for 30 minutes. The culture medium was removed by aspiration and dried for 10 minutes. An aliquot of 200 µl DMSO was added to dissolve the formazan crystals formed from the reduction of MTT reagent by the mitochondrial reductase of live cells. To determine the amount of formazan formed, three aliquots (100 µl each) of the mixture from each well of cells were transferred in three separate wells of a 96-well microtiter plate (Falcon) and the absorbance at 570 nm was measured using Envision Multilabel plate reader (PerkinElmer, USA). The cell viability was compared as a percentage of DMSO treated and untreated astrocytes (0 hr) for CP-316819 and DAB treated astrocyte cultures, respectively.

### LDH assay

The LDH assay was performed in dark according to the manufacturer's protocol (Promega, USA). Briefly, after induction of hypoxia for different time points, 50 µl of culture medium from each well of the culture plate containing astrocytes was transferred to 96 well-micro titre plates (Falcon). A 100 µl of LDH assay mixture containing equal volume of LDH Assay Substrate solution, LDH Assay Dye solution and LDH Assay Cofactor was then added to each well. The reaction was allowed to proceed at room temperature for 30 minutes in dark and was stopped by adding 50 µl of 1 mM acetic acid. The absorbance of whole mixture was measured in triplicate at a wavelength of 490 nm using Envision Multilabel plate reader (PerkinElmer, USA). The release of LDH was calculated as a percentage of DMSO treated and untreated astrocytes (0 hr) for CP-316819 and DAB treated astrocyte cultures, respectively.

### Monitoring live and dead cells by calcein-AM and EthD-1 staining

Calcein acetoxymethyl (calcein-AM) and ethidium homodimer-1 (EthD-1) were used to visualize live and damaged astrocyte respectively after induction of hypoxia. The disrupted cell membranes of damaged astrocytes allow entry of EthD-1, which upon binding to nucleic acids, produces red fluorescence (excitation wavelength, ∼528 nm; emission wavelength, ∼617 nm). While the membrane-permeable calcein-AM is converted by the intracellular esterases of live cells only to form intensely fluorescent calcein (excitation wavelength, ∼494 nm; emission wavelength, ∼517 nm).

In brief, after induction of hypoxia, the astrocyte cultures were washed twice with 1× PBS and stained with calcein-AM and EthD-1 (final concentrations: 2 µM and 5 µM respectively) (Invitrogen, USA) dissolved in DMEM without phenol red and incubated at 37°C for 30 minutes in a 5% CO_2_ incubator. After staining, the cells were washed twice with 1× PBS. Live and dead cells were visualized (5–6 images for each well) using a fluorescence microscope (Leica DMI6000B) and the number of viable and dead cells was counted using the image J software. The ratios of live to dead cells obtained from each of the groups were compared.

### Statistical analysis

Densitometry analyses for the quantification of the bands on immunoblots were performed using Image J software. Statistical analyses were performed with the Statistical Package for Social Sciences for Windows, version 16 (SPSS, Inc, Chicago, Illinois, USA). The data were reported as the mean ±SD and the statistical significance were determined by parametric procedure as student's t-test (two-tailed) and one-way analysis of variance (ANOVA) followed by Tukey's *post hoc* test (for multiple comparisons). p<0.05 was considered statistically significant for all experiments.

## Results

### Stroke induction

Among the rats used in the current study, 4 were excluded from the analysis due to lack of observed behavioral changes during stroke induction. Total mortality was 1/28 due to severe stroke. Some post-stroke weight loss was observed in the 24 hours recovery groups.

### Glycogen is accumulated in contralateral hemisphere during ischemic stroke

**Figure 1:**
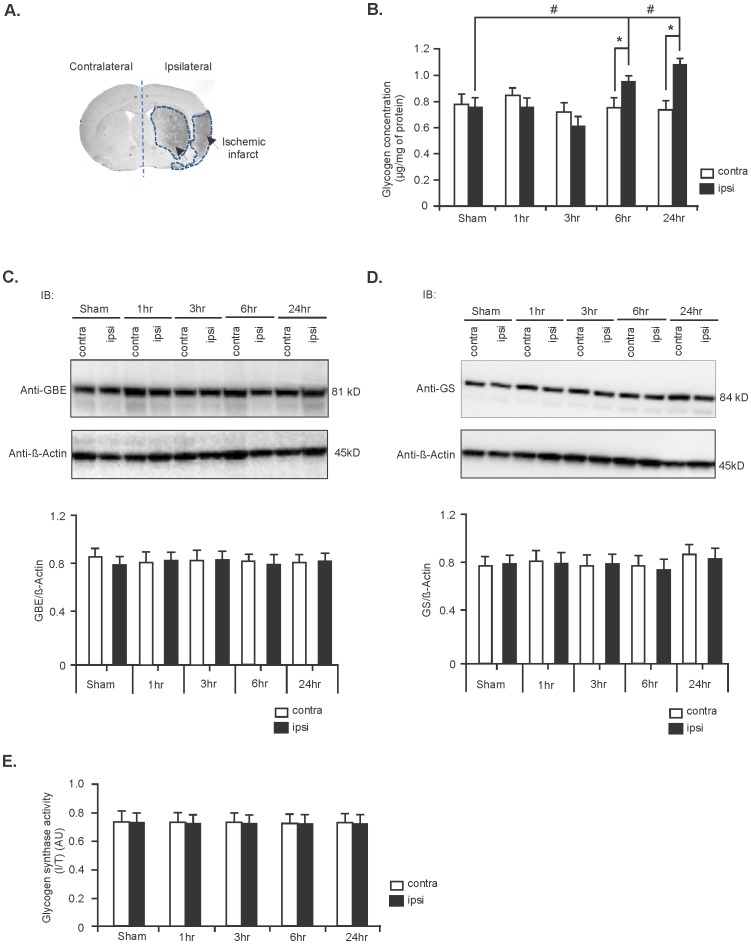
Glycogen is accumulated in stroke-affected brain. (**A**) A representative sample image generated from an unstained section collected between 1.2 mm to −2.8 mm bregma from an ET-1-induced stroke rat brain. Stroke-induced damage is shown to be located predominately around the site of ET-1-induced vasoconstriction, referred to as the ‘core' ischemic infarct (arrow sign) in the ipsilateral hemisphere. (**B**) Glycogen concentration in contralateral and ipsilateral hemispheres of control (sham) and stroke brains. Values are expressed as mean ±SD, n = 5 rats for each group. ^#^ *P* and ^*^ *P*<0.05 compared to sham ipsilateral and stroke contralateral hemispheres, respectively. Expression levels of GBE (**C**) and GS (**D**) in contralateral and ipsilateral hemispheres of sham and stroke rat brains between 1 hr and 24 hr. Expression levels were normalized against β-Actin (middle panel). Quantification of this data is shown in the lower panel. Data are mean ±SD, n = 5. (**E**) GS activity in sham and stroke rat brains at different time points post-stroke. Data are represented as mean ±SD; n = 5. (^*^ *P*  =  Student's t-test and ^#^ *P* =  one-way ANOVA followed by Tukey's *post hoc* test).

We first assessed the metabolic fate of glycogen in ischemic stroke-affected rat brains. For this, we measured the level of glycogen in control (sham) and ischemic stroke brains in both the contralateral and ipsilateral hemispheres that underwent ET-1-induced stroke after 1, 3, 6 and 24 hours of recovery. We found that glycogen levels were increased in the ipsilateral (stroke-affected) hemispheres compared with that of the contralateral hemispheres between 6 and 24 hours after stroke induction (25% and 39% increase, respectively; n = 5, ^*^ *P*<0.05; Figure 1B) as well ipsilateral hemisphere of sham operated controls (23% and 34% increase, respectively; n = 5, ^#^ *P*<0.05; Figure 1B).

The two key enzymes involved in glycogen synthesis are GBE and GS. We therefore monitored the expression level of these two enzymes after stroke at different time intervals. We found that the expression levels of both enzymes remained unchanged in the contralateral and ipsilateral hemispheres of stroke brains in comparison to sham controls (Figure 1C and D respectively, n = 5). In addition, the activity of GS was unaltered in the stroke-affected rat brains in comparison to sham controls (Figure 1E, n = 5).

### Expression of GDE, and activity of GP and PKA are suppressed in ischemic stroke

**Figure 2:**
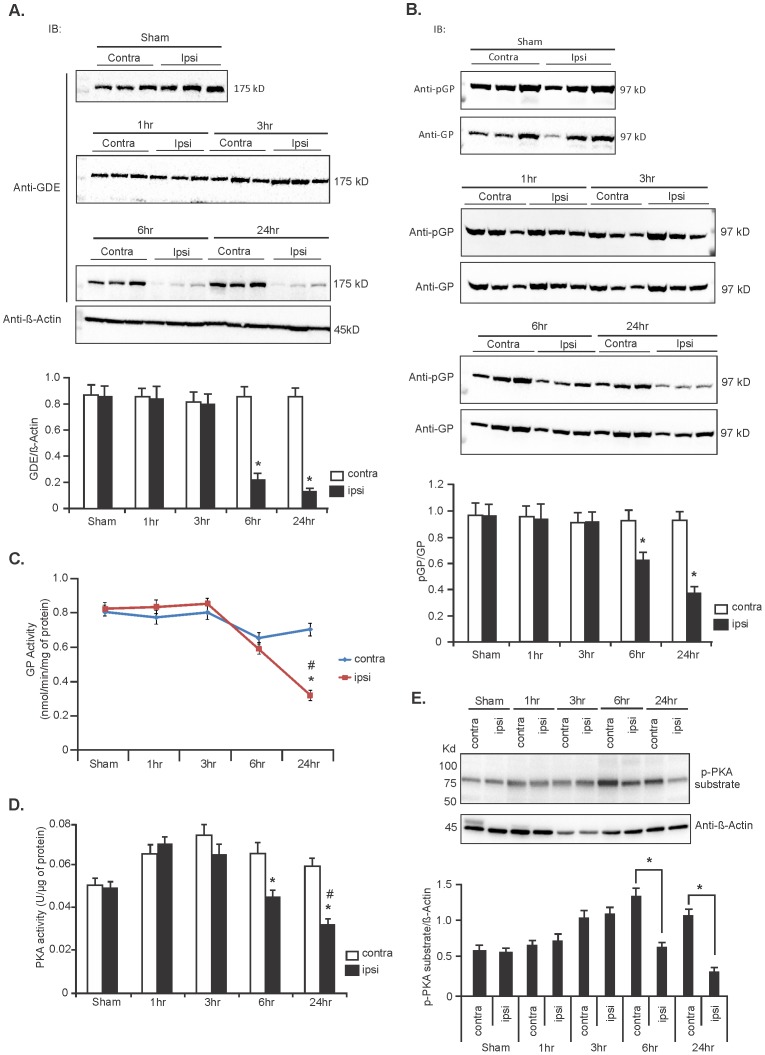
Expression levels of GDE, and activity of GP and PKA are decreased during ischemic stroke. (**A**) Representative immunoblot showing expression levels of GDE in contralateral and ipsilateral hemispheres of sham and stroke brains at different time points following stroke induction. Expression level of GDE was normalized against β-Actin protein. Lower panel: Quantification data of GDE expression level. Data are represented as mean ±SD, n = 6; ^*^ *P*<0.05 vs contralateral hemispheres (6 hr and 24 hr). (**B**) Representative immunoblot of phosphorylation level of GP in sham and stroke rat brains. Immunoblot analysis data (lower panel) as mean ±SD, n = 6, ^*^ *P*<0.05 vs contralateral hemispheres of stroke brains (6 hr and 24 hr). (**C**) GP enzyme activity in sham and stroke rat brains. Data are mean ±SD, n = 6 rats for each group. ^#^ *P* = 0.001 vs sham ipsilateral and ^*^ *P* = 0.003 vs stroke contralateral hemisphere (24 hr). (**D**) PKA activity in sham and stroke brains [mean ±SD, n = 6;^ #^ *P* = 0.003 (24 hr) vs sham ipsilateral and ^*^ *P* = 0.004 (6 hr) and 0.003 (24 hr) vs stroke contralateral hemispheres]. (**E**) Representative immunoblot of p-PKA substrate of sham and stroke brains. Phosphorylation level was normalized against β-Actin protein [mean ±SD, n = 5; ^*^ *P* = 0.003 (6 hr) and 0.002 (24 hr)]. (^*^ *P* =  Student's t-test and ^#^ *P* =  one-way ANOVA followed by Tukey's *post hoc* test).

The enzymes essential for glycogen degradation are GDE and the brain isoform of GP. GDE converts α-1,6-glycosidic linkages in glycogen to α-1,4-glycosidic linkages for further breakdown by GP [35]. We therefore investigated the expression level of GDE and activity of GP various times following initiation of the stroke. Immunoblot analyses of sham and stroke- affected rat brains were performed at 1, 3, 6 and 24 hours after stroke induction. Immunoblot analyses revealed that the expressions of GDE in the stroke-affected brain at early time points (1 and 3 hr) were similar to that of the sham controls (Figure 2A). GDE expression, however, was significantly lower in the ipsilateral hemispheres compared with that of the contralateral and sham controls (67% and 77% decrease in stroke contralateral hemisphere, respectively, Figure 2A, lower panel; n = 6, ^*^ *P*<0.05) at later time points. Analysis of GP phosphorylation level at Ser-14 (pGP) by immunoblot revealed a significant decrease of phosphorylation in the stroke ipsilateral hemispheres compared with that of the contralateral hemispheres and sham controls at 6 and 24 hours time points (Figure 2B and lower panel; n = 6, ^*^ *P*<0.05). Measurement of GP activity further confirmed a decrease in phosphorylase activity in the ipsilateral hemispheres 2 hours post-stroke compared with that of the contralateral hemispheres (58% reduction, n = 6, ^*^ *P* = 0.003; Figure 2C) and ipsilateral hemispheres of sham operated controls (61.5% reduction, n = 6,^ #^ *P* = 0.001; Figure 2C). GP is primarily regulated by phosphorylation at Ser-14 by phosphorylase kinase (PhK) which is activated by cAMP-dependent protein kinase A (PKA) [6], [36]. We therefore measured PKA activity in sham and stroke-induced rat brains. PKA activity was significantly reduced in the ipsilateral hemisphere compared with that of the contralateral hemisphere in stroke rat brains at 6 and 24 hours post-stroke [32% and 48.3% reduction, respectively, n = 6; ^*^ *P* = 0.004 (6 h) and 0.003 (24 h); Figure 2D] and sham controls [36% reduction vs sham ipsilateral hemisphere, n = 6; ^#^ *P* = 0.003 (24 h); Figure 2D]. Immunoblot data with a p-PKA substrate antibody that detects all PKA-phosphorylated motifs in sham and stroke rat brain homogenates further indicated a down-regulation of p-PKA immunoreactivity (p-PKA substrate blot) in the ipsilateral hemispheres in comparison to the contralateral hemispheres in stroke brains [Figure 2E, n = 5, ^*^ *P* = 0.003 (6 h) and 0.002 (24 h)]. We further noticed that the contralateral hemispheres of stroke rat brains (3, 6 and 24 hr) showed increased PKA activity and p-PKA substrate immunoreactivity compared with either hemispheres of the sham rat brains (Figure 2D and E), therefore, we further analyzed PKA activity of contralateral and ipsilateral regions of sham rat brains after saline infusion at different time points. Though, we found increased p-PKA substrate immunoreactivity and PKA activity in both hemispheres of sham rat brains at later time points compared with that of the earlier time points, the changes were statistically not significant among the groups (Figure S3 A and B in Supporting Information S1).

### The mRNA expression level of glycogen metabolizing enzymes in sham and ischemic stroke rat brains

**Figure 3:**
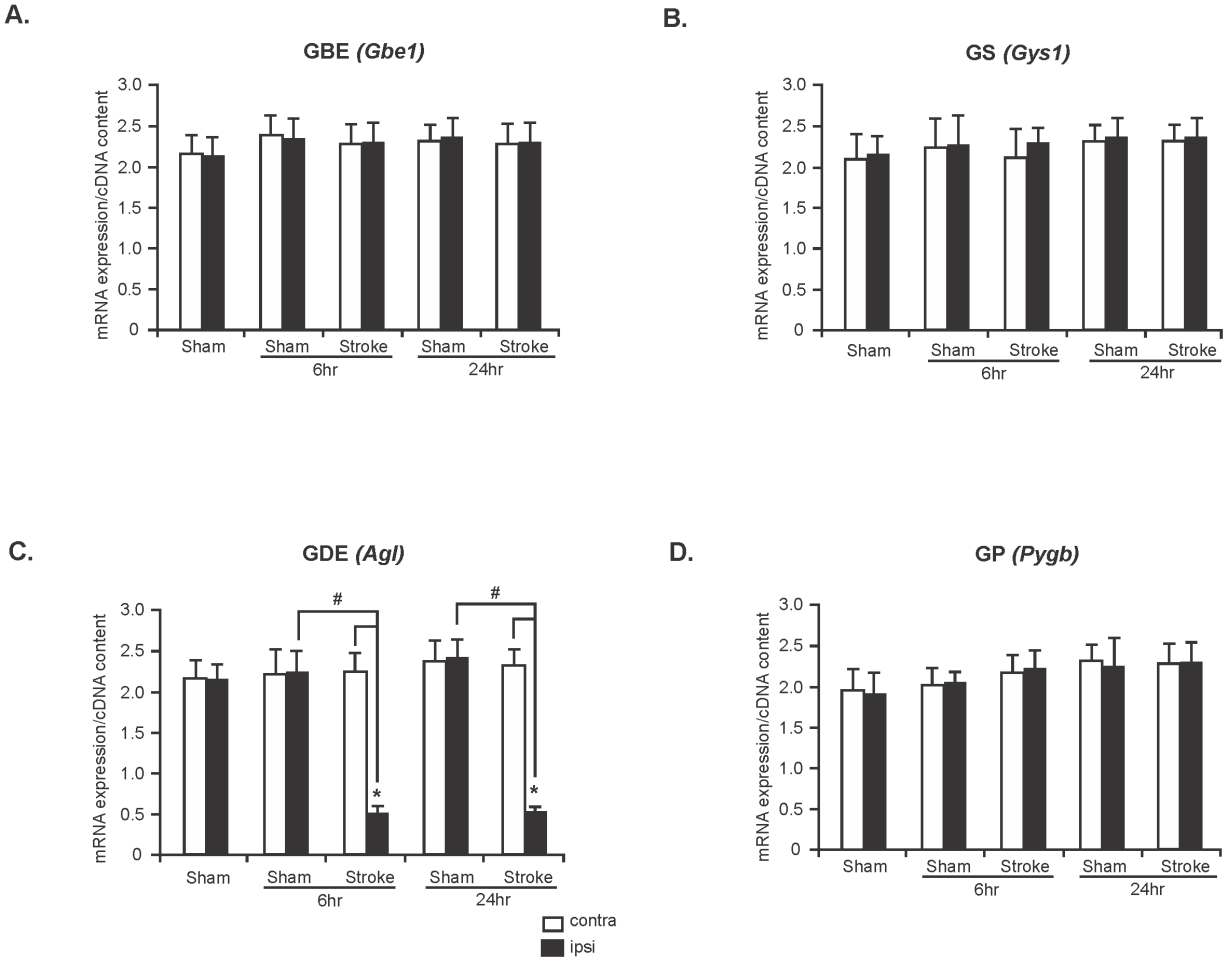
Reduced GDE mRNA expression in ischemic stroke. mRNA expression of GBE (*Gbe1*) (**A**), GS (*Gys1*) (**B**), GDE (*Agl*) (**C**) and GP (*Pygb*) (**D**) in contralateral and ipsilateral hemispheres of sham and stroke rat brains. Data are presented as mean ±SD, n  =  5 for each group. *Agl* analysis: ^*^ *P*<0.05 vs contralateral hemispheres of stroke rat brains (6 and 24 hr) and ^#^ *P*<0.05 vs ipsilateral hemispheres of sham rat brains of 6 hr and 24 hr groups. (^*^ *P* =  Student's t-test and ^#^ *P* =  one-way ANOVA followed by Tukey's *post hoc* test).

To assess whether ischemic insult alters the mRNA expression level of genes involved in glycogen metabolism we measured mRNA expression level of *Gbe1*, *Gys1*, *Agl* and *Pygb* in the contralateral and ipsilateral hemispheres of sham and stroke-affected rat brains. Real-Time RT-PCR data revealed that mRNA expression levels of *Gbe1*, *Gys1*, and *Pygb* remained unchanged in contralateral and ipsilateral hemispheres of both sham and stroke rat brains at 6 and 24 hours time points (Figure 3A, B and D, n = 5). However, the level of mRNA expression of *Agl* was significantly decreased in the ipsilateral hemispheres compared with that of the contralateral hemispheres and sham controls at 6 and 24 hours (73% and 72% reduction, respectively, Figure 3C, n = 5; ^*^ *P* and ^#^ *P*<0.05).

### Hypoxia induces accumulation of glycogen in astrocytes

**Figure 4:**
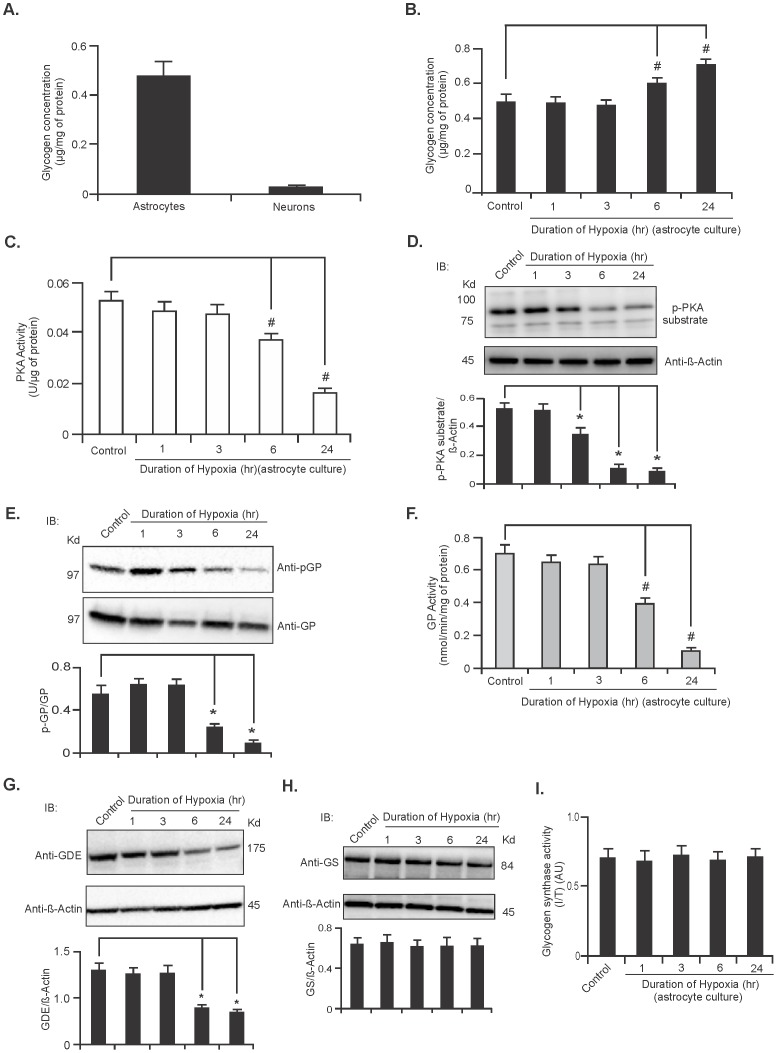
Glycogen is accumulated in astrocytes during hypoxia. (**A**) Glycogen levels in rat primary cerebellar astrocytes and cortical neurons. Data are expressed as mean ±SD of 5 independent cultures. (**B**) Glycogen levels in rat cerebellar astrocytes kept under hypoxic conditions for 0 (control), 1, 3, 6 and 24 hr as described in the Materials and Methods section. Glycogen levels were measured following 24 hr incubation in normoxic condition. Values are expressed as mean ±SD, n = 5; ^#^ *P*<0.05 vs control astrocytes. (**C**) PKA activity in astrocytes exposed to hypoxic conditions. (Data are mean ±SD, n = 5; ^#^ *P*<0.05 compared with control astrocytes). (**D**) Representative immunoblot of p-PKA substrate immunoblot analysis in astrocytes maintained in hypoxic conditions for the indicated time periods. Phosphorylation levels were normalized against β-Actin protein, the analysis is shown in the lower panel (mean ±SD, n = 5; ^*^ *P*<0.05). (**E)** Representative immunoblot of pGP in astrocytes incubated under hypoxic conditions. Quantification of phosphorylation levels was performed using the total level of GP protein. Quantification data (lower panel) are represented as mean ±SD, n = 5; ^*^ *P*<0.05. (**F**) GP activity in astrocytes during hypoxia. Data are mean ±SD, n = 5; ^#^ *P*<0.05 vs control astrocytes. (**G**) Representative immunoblot showing expression level of GDE in astrocytes exposed to hypoxic condition for indicated time points. Expression of GDE was normalized against β-Actin protein. Data are represented as mean ±SD, n = 5;^ *^ *P*<0.05 vs control astrocytes. GS protein expression (**H**) and GS enzymatic activity (**I**) in hypoxic astrocytes. Data are represented as mean ±SD, n = 5. (^*^ *P* =  Student's t-test and ^#^ *P* =  one-way ANOVA followed by Tukey's *post hoc* test).

To investigate the effect of hypoxia on glycogen metabolism *in vitro* we cultured rat primary cerebeller astrocytes and cortical neurons and measured total amount of glycogen present in these two types of cells. Glycogen was mainly localized to astrocytes while neurons contained very little or no glycogen (Figure 4A, n = 5) confirming previous studies [37]. Next, we induced hypoxia in rat cerebeller astrocytes for 1, 3, 6 and 24 hours and monitored its effect on glycogen level. The cerebellar astrocyte cultures exposed to hypoxia for 6 and 24 hours showed significantly higher amounts of accumulated glycogen compared with that of the control (0 hr) astrocytes (Figure 4B, n = 5; ^*^ *P*<0.05) suggesting decreased breakdown of astrocytic glycogen during hypoxia. To explore the potential effect of PKA in this pathway we further investigated PKA's activity in hypoxic astrocytes by enzymatic assay and by immunoblot using an anti-p-PKA substrate antibody. As shown in Figure 4C and D PKA activity was significantly reduced in astrocytes exposed to hypoxic condition for 6 and 24 hours (n = 5; ^*^ *P*<0.05). Since GP is essential to glycogen metabolism and is also an indirect downstream target of PKA, we therefore measured GP activity by both phosphoimmunoblot and enzymatically in hypoxic astrocytes. These experiments showed a significant decrease in GP activity in hypoxic astrocytes at 6 and 24 hours periods in comparison to that of the control astrocytes (Figure 4E and F, respectively, n = 5; ^*^ *P*<0.05). The expression level of GDE was decreased in hypoxic astrocytes (Figure 4G, n = 5; ^*^ *P*<0.05) while both expression and activity of GS remained unchanged (Figure 4H and I, respectively, n = 5).

### Inhibition of PKA activity leads to increase glycogen accumulation in cultured astrocytes

**Figure 5:**
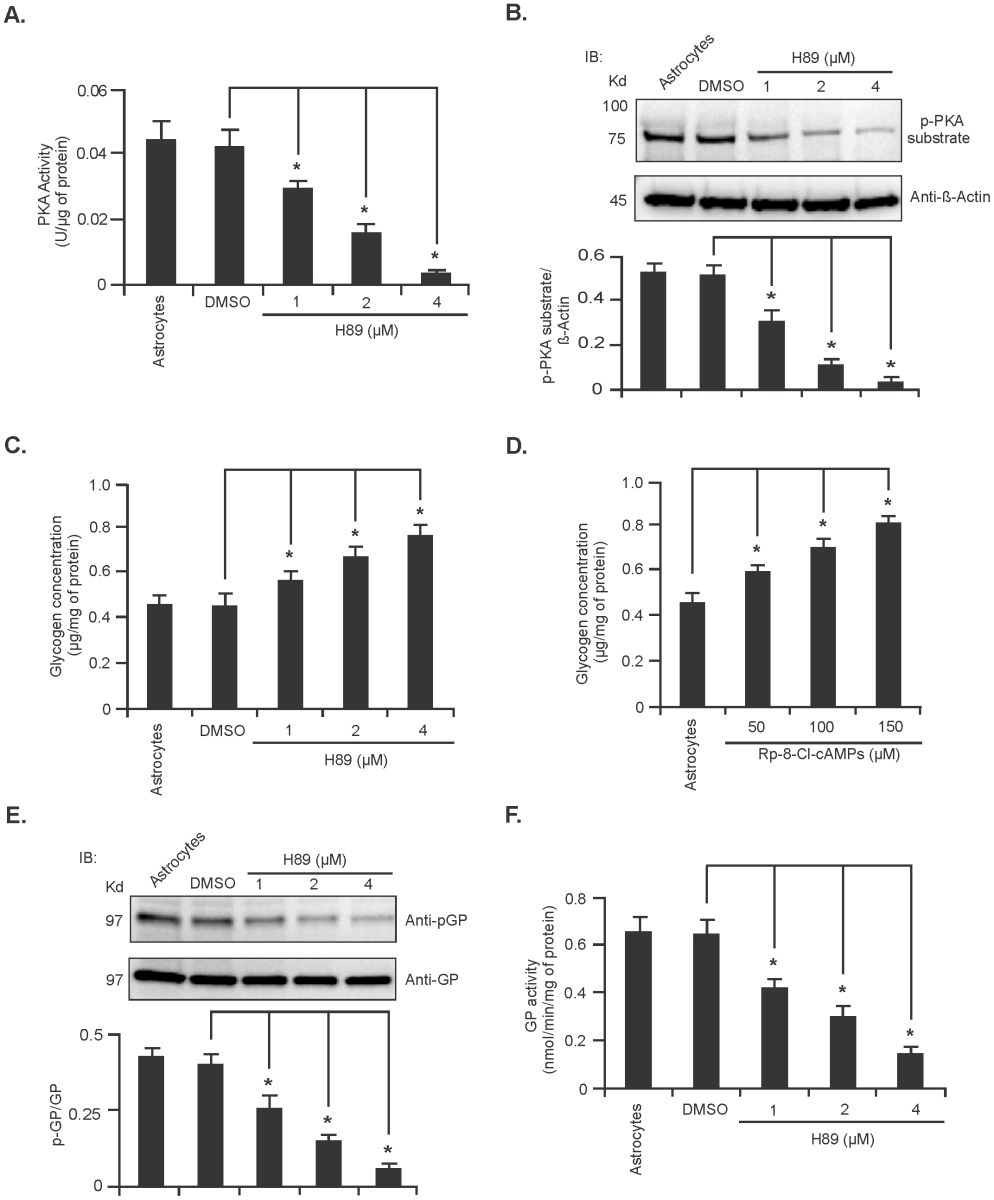
Inhibition of PKA activity in cultured cerebellar astrocytes increases glycogen accumulation. (**A**) PKA activity in cerebellar astrocytes treated with different concentrations of H89 for 24 hr [mean ±SD, n = 5; ^*^ *P*<0.05 compared with DMSO (vehicle) treated astrocytes]. (**B**) Representative immunoblot of p-PKA substrate in astrocytes treated with either DMSO or H89 for 24 hr. Phosphorylation level was normalized against β-Actin protein, the analysis is shown in the lower panel (n = 5; ^*^ *P*<0.05). Glycogen levels in astrocytes treated with H89 (**C**) and Rp-8-Cl-cAMPs (**D**) (mean ±SD, n = 5; ^*^ *P*<0.05). (**E**) Immunoblot of pGP in H89 treated astrocytes. Quantification, shown in the lower panel, was performed using total GP protein. Data are represented as mean ±SD, n = 5; ^*^ *P*<0.05. (**F**) GP activity in H89 treated astrocytes. Data are mean ±SD, n = 5; ^*^ *P*<0.05 vs DMSO treated astrocytes. (^*^ *P* =  Student's t-test).

To confirm the direct involvement of PKA in the regulation of glycogen metabolism, rat primary cerebellar astrocytes were treated with 1, 2 and 4 µM of the PKA inhibitor H89 for 24 hours. Both PKA activity measurements demonstrated that PKA activity was decreased in a dose dependant manner upon H89 treatment (Figure 5A & B respectively, n = 5; ^*^ *P*<0.05). Next, we measured the level of glycogen in astrocytes treated with H89. As shown in Figure 5C astrocytes treated with H89 contained significantly increased amounts of glycogen in comparison to that of the vehicle (DMSO) treated astrocytes (Figure 5C, n = 5; ^*^ *P*<0.05). For further confirmation, we determined the glycogen level in astrocytes treated with a different PKA inhibitor, Rp-8-Cl-cAMPs using concentrations ranging from 50 to 150 µM for 24 hours, and similarly found that the level of glycogen was increased significantly in astrocytes upon treatment with the PKA inhibitor in a dose dependent manner (Figure 5D, n = 5; ^*^ *P*<0.05). We next investigated whether decreased PKA activity could also decrease GP enzyme activity. As shown in Figures 5E and F, treatment of astrocytes with H89 resulted in decreased GP phosphorylation (Ser-14) and enzyme activity, respectively (n = 5; ^*^ *P*<0.05). Decreased PKA or GP activity however, did not alter the expression of GDE or the activity of GS (Figure S4A and B in Supporting Information S1). Taken together, these data suggest that inhibition of PKA activity leads to the accumulation of glycogen in astrocytes by decreasing GP activity but has no effect on the activity of GS or expression of GDE.

### Effects of elevated glycogen on the viability of astrocytes during hypoxia

**Figure 6:**
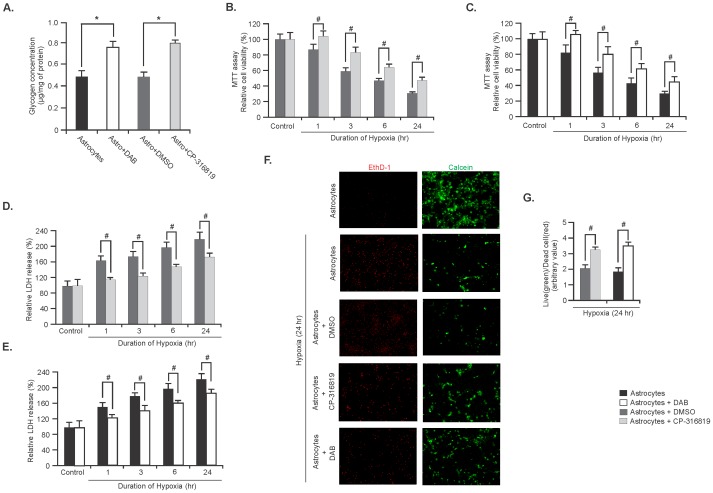
Elevated glycogen levels protect astrocytes during hypoxia. (**A**) Glycogen levels in astrocytes treated with either DAB (1 mM) or CP-316819 (10 µM) or DMSO for 24 hr. Data are presented as mean ±SD, n = 5; ^*^ *P*<0.05. MTT assay showing cell viability in astrocytes treated with either CP-316819 (**B**) or DAB (**C**) for 24 hr and exposed to hypoxia for indicated time points. (Data are mean ±SD, n = 5; ^#^ *P*<0.05). LDH assay in astrocytes treated with either CP-316819 (**D**) or DAB (**E**) for 24 hr and exposed to hypoxia. (Data are mean ±SD, n = 5; ^#^ *P*<0.05). (**F**) Representative images of live (green) and dead (red) cells in astrocytes exposed to hypoxia for 24 hr. Astrocytes were treated with CP-316819, DMSO or DAB for 24 hr prior to hypoxia. (**G**) Ratio of live to dead cells in astrocytes treated with either CP-316819 or DAB and exposed to hypoxia for 241hr. Data are mean ±SD, n = 5; ^#^ *P*<0.05. (^*^ *P* =  Student's t-test and ^#^ *P* =  one-way ANOVA followed by Tukey's *post hoc* test).

In the final experiments we assessed the effect of elevated glycogen in astrocytes during hypoxic insults. For this astrocyte cultures were treated with GP inhibitors, CP-316819 or DAB to increase glycogen content. Glycogen levels in astrocytes were significantly increased following incubation of the astrocytes with the two different GP inhibitors for 24 hours (Figure 6A, n = 5; ^*^ *P*<0.05). Astrocyte viability following GP inhibitors treatment and hypoxia induction were monitored by MTT and LDH assay, and by the live/dead cell staining method.

The MTT assay data showed that cell viability was significantly higher in either CP-316819 or DAB treated astrocytes compared with that of the DMSO treated or control astrocytes (untreated) respectively at each time points of hypoxia (Figure 6B and C, respectively n = 5; ^#^ *P*<0.05). The LDH assay revealed that CP-316819 or DAB treated astrocytes had significantly decreased level of LDH released in the culture medium compared with that of the DMSO treated or control (untreated) astrocytes under hypoxic conditions (Figure 6D and E, respectively, n = 5; ^#^ *P*<0.05). Viability of the astrocytes following 24 hours of hypoxia by calcein AM and EthD-1 showed that both CP-316819 and DAB treated astrocytes contained more viable cells (green) compared with DMSO treated or untreated astrocytes, respectively (Figure 6F). Moreover, the live to dead cell ratios revealed that CP-316819 or DAB treated astrocytes had significantly higher number of live cells compared with the control groups of astrocytes (Figure 6G, n = 5; ^#^ *P*<0.05).

## Discussion

During ischemic stroke extracellular potassium levels rise within a few seconds of ischemic insults, which is preceded by rapid Ca^2+^ influx and a fall in ATP levels, triggering cascades of numerous ionic and biochemical events that initiate cellular demise [2], [20], [38]. The ability of ischemic tissue to recover normal functions requires a sustained supply of energy to re-establish the electrolyte imbalances between the intracellular and extracellular compartments that arise during the insult [2], [4]. Brain glycogen has been reported to serve as an endogenous source of metabolic energy during periods of ischemia and can be targeted as a therapeutic approach to cerebral ischemia [39], [40].

In the present study, we found an increase in the level of glycogen in the stroke-affected brain between 6 and 24 hours post-stroke (Figure 1B) suggesting impaired glycogen metabolism. To determine the signaling pathways involved in this process, we measured the expression level and activity of glycogen synthesizing enzymes GBE and GS. These enzymes are responsible for elongation of newly synthesized glycogen molecules by forming *α*-1,4-glycosidic linkages with UDP-glucose as the glucosyl donor and *α*-1,6-glycosidic linkages. We found no changes in the expression level of GBE, nor changes in the expression and activity of GS (Figure 1C, D and E), suggesting that these enzymes are not of the direct cause for glycogen accumulation in the ischemic brain.

Enzymatic cleavage of glycogen to generate glucose-1-phosphate during scarcity of glucose is exclusively catalyzed by GP in conjunction with GDE, the enzyme essential for removing branched glucose chains in the glycogen particle. Decreased activity and expression level of these enzymes would contribute to the accumulation of glycogen. We clearly showed a decreased GP activity in stroke-affected rat brains at 6 and 24 hours post-stroke (Figure 2B and C) that would contribute to the increased glycogen amount. In addition, we unexpectedly found significantly reduced expression of GDE in the ischemic stroke brain at these time points (Figure 2A). Reduced GDE expression was further validated by reduced mRNA level of GDE gene, *Agl* in the stroke brain (Figure 3C). The mechanism behind this reduced GDE mRNA expression has never been reported but suggests that the stroke-induced cellular environment includes a negative regulator of GDE gene expression. This observed GDE expression, would lead to incomplete glycogen breakdown since GP cannot degrade glycogen beyond the *α*-1,6 branch points. Therefore, only 30% of the glycogen particle could be degraded by GP [41] before resynthesis could take over again. Furthermore, given that GP is also partially inactive, the amount of available glucose derived from glycogen breakdown would be significantly blunted in the stroke-affected brain.

Our investigation in hypoxic astrocytes confirmed these data with increased glycogen (Figure 4B), decreased PKA and GP activity (Figure 4C, D, E and F) and decreased GDE expression (Figure 4G), suggesting similar signaling pathways are involved in the regulation of glycogen metabolism during ischemic stroke and hypoxia. During a shortage of cellular ATP, β_1_ and/or β_2_ adrenergic receptors are activated and trigger a cascade of signal transduction activating adenylyl cyclase (AC) [42]. Active AC converts ATP to cAMP leading to PKA activation. Active PKA phosphorylates and activates PhK, which in turn, activates GP by phosphorylating Ser-14 leading to glycogen breakdown [43]. PhK can also be activated by intracellular Ca^2+^ and directly phophorylates GP, converting it from inactive (GP*b*) to active (GP*a*) form [36]. The decreased PKA activity in the stroke-affected brain between 6 and 24 hours (Figure 2D and E) suggests insensitivity of the adrenoreceptors to the energy-deficient state. This insensitivity might be the result of increased expression of β-arrestin-1 during cerebral ischemia [44], which binds G-protein receptor kinase (GRK)-mediated phosphorylated form of the adrenoreceptors, preventing coupling of the receptor with G-protein, and also recruits phosphodiesterases that mediate degradation of cAMP [45]–[47]. Uncoupling of the receptor also causes receptor internalization through β-arrestin and clarithin depended pathway and reduces the number of functional receptors available in the cell membrane [47]–[49]. Low levels of cAMP might also be responsible for decreased PKA and adrenoreceptor activity as both are regulated by cAMP [42], [50], [51]. We hypothesized that the decreased activity of PKA contributes to the accumulation of glycogen in stroke brain and in the primary cerebellar astrocytes (Figure 5C and D), possibly by suppressing activity of GP (Figure 5E and F).

Increased glycogen accumulation following induction of stroke could also be caused by glycogen supercompensation, a phenomenon that has been observed in the skeletal muscle following exercise and in the brain [52]. The mechanism, in muscle at least, is caused by activation of GS and inactivation of GP [53] but the mechanism in the brain is unknown. Glycogen supercompensation may be a protective measure taken by the affected tissue in preparation for the next bout of metabolic stress [23]. Therefore, the increased glycogen observed during hypoglycaemia and also in this study of ischemia/hypoxia could be considered similar, as they both occur following metabolic stress. Furthermore, elevated glycogen can also protect astrocytes during hypoxia as we demonstrated (Figure 6) and as shown previously [13], [54].

**Figure 7:**
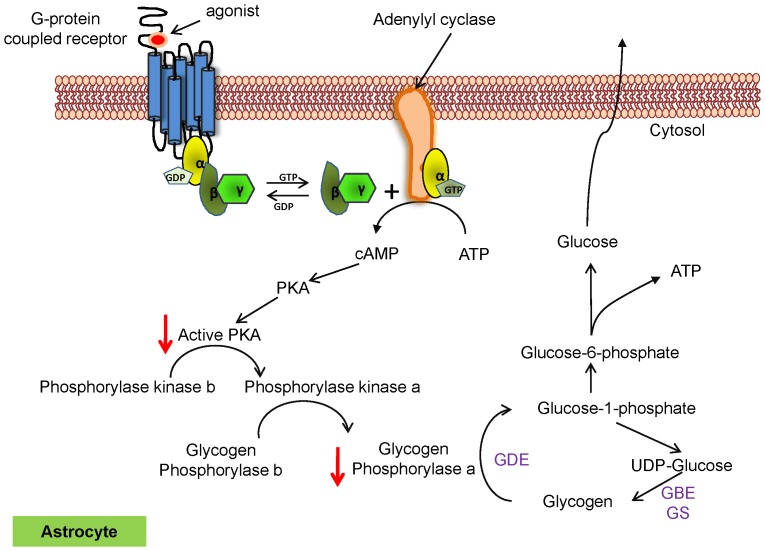
Signaling model of PKA's involvement in impaired glycogen metabolism during ischemic stroke. Within astrocytes under physiological conditions, the binding of an agonist to its receptor leads to the activation of G-proteins (α, β and γ subunits), which in turn activate adenylyl cyclase (AC). Active AC then converts ATP to cAMP that binds to the regulatory subunits of tetrameric PKA. This releases free catalytic subunits that phosphorylate phosphorylase kinase b to convert it to active phosphorylase kinase a. Active phosphorylase kinase then phosphorylates GP*b* to GP*a* initiating glycogen breakdown to provide energy for associating neurons [4], [59]. Inhibition of PKA activity during ischemic conditions, however suppresses this downstream signaling cascade that is essential for glycogen metabolism leading to the accumulation of glycogen in astrocytes in the stroke-affected brain.

A schematic diagram (Figure 7) based on the data we found indicates that inhibition of PKA activity decreases GP activity resulting in impaired breakdown of glycogen. Manipulation of these pathways may be a beneficial strategy for optimization of brain glycogen metabolism and maintenance of glucose homeostasis during ischemic stroke. Beneficial effects of pharmacological manipulation of cAMP/PKA-mediated pathways have been recently documented in other diseases including diabetes and cancer [55], [56], suggesting that this approach should be investigated in animal models of ischemic stroke to test the efficacy as a therapeutic intervention. Furthermore, compounds that increase cAMP generation in astrocytes might be useful in providing metabolic support to neurons during conditions such as glucose deprivation and intense neuronal activity by mobilizing glycogen content in astrocytes. In support of this, soluble adenylyl cyclase (sAC) has been reported to propagate cellular cascades to meet metabolic demands by initiating breakdown of glycogen. HCO_3_^−^ mediated activation of sAC increases cAMP concentration [57], leading to the breakdown of glycogen [58] thereby providing an alternative energy source to neurons. Recently, it has been demonstrated that sAC is highly expressed in astrocytes and activation of this enzyme increases glycogen breakdown via increased intracellular cAMP to provide energy to neurons [42].

In summary, we demonstrate that decreased PKA activity along with decreased GP activity and GDE expression induce impaired glycogen metabolism in ischemic stroke rat brain. To the best of our knowledge this is the first comprehensive study of the regulation of metabolic enzymes in the ischemic stroke brain projecting new light in glycogen metabolism and may provide new therapeutic intervention strategies to prolong cellular activity and minimize post-ischemic damage.

## Supporting Information

Supporting Information S1 **Supplement information and supporting figures.**
(DOC)

## References

[1] Lo EH (2010) Degeneration and repair in central nervous system disease. Nat Med 16: 1205–1209. 21052074 10.1038/nm.2226 PMC3985732

[2] Moskowitz MA, Lo EH, Iadecola C (2010) The science of stroke: mechanisms in search of treatments. Neuron 67: 181–198. 20670828 10.1016/j.neuron.2010.07.002 PMC2957363

[3] Lipton P (1999) Ischemic cell death in brain neurons. Physiol Rev 79: 1431–1568. 10508238 10.1152/physrev.1999.79.4.1431

[4] Rossi DJ, Brady JD, Mohr C (2007) Astrocyte metabolism and signaling during brain ischemia. Nat Neurosci 10: 1377–1386. 17965658 10.1038/nn2004 PMC8906499

[5] Roach PJ (2002) Glycogen and its metabolism. Curr Mol Med 2: 101–120. 11949930 10.2174/1566524024605761

[6] Roach PJ, Depaoli-Roach AA, Hurley TD, Tagliabracci VS (2012) Glycogen and its metabolism: some new developments and old themes. Biochem J 441: 763–787. 22248338 10.1042/BJ20111416 PMC4945249

[7] Kong J, Shepel PN, Holden CP, Mackiewicz M, Pack AI, et al (2002) Brain glycogen decreases with increased periods of wakefulness: implications for homeostatic drive to sleep. J Neurosci 22: 5581–5587. 12097509 10.1523/JNEUROSCI.22-13-05581.2002 PMC6758203

[8] Suh SW, Bergher JP, Anderson CM, Treadway JL, Fosgerau K, et al (2007) Astrocyte glycogen sustains neuronal activity during hypoglycemia: studies with the glycogen phosphorylase inhibitor CP-316,819 ([R-R*,S*]-5-chloro-N-[2-hydroxy-3-(methoxymethylamino)-3-oxo-1-(phenylmethyl)pro pyl]-1H-indole-2-carboxamide). J Pharmacol Exp Ther 321: 45–50. 17251391 10.1124/jpet.106.115550

[9] Gibbs ME, Anderson DG, Hertz L (2006) Inhibition of glycogenolysis in astrocytes interrupts memory consolidation in young chickens. Glia 54: 214–222. 16819764 10.1002/glia.20377

[10] Shulman RG, Hyder F, Rothman DL (2001) Cerebral energetics and the glycogen shunt: neurochemical basis of functional imaging. Proc Natl Acad Sci U S A 98: 6417–6422. 11344262 10.1073/pnas.101129298 PMC33483

[11] Tekkok SB, Brown AM, Westenbroek R, Pellerin L, Ransom BR (2005) Transfer of glycogen-derived lactate from astrocytes to axons via specific monocarboxylate transporters supports mouse optic nerve activity. J Neurosci Res 81: 644–652. 16015619 10.1002/jnr.20573

[12] Rahman B, Kussmaul L, Hamprecht B, Dringen R (2000) Glycogen is mobilized during the disposal of peroxides by cultured astroglial cells from rat brain. Neurosci Lett 290: 169–172. 10963890 10.1016/s0304-3940(00)01369-0

[13] Brown AM, Sickmann HM, Fosgerau K, Lund TM, Schousboe A, et al (2005) Astrocyte glycogen metabolism is required for neural activity during aglycemia or intense stimulation in mouse white matter. J Neurosci Res 79: 74–80. 15578727 10.1002/jnr.20335

[14] Hertz L, Gibbs ME (2009) What learning in day-old chickens can teach a neurochemist: focus on astrocyte metabolism. J Neurochem 109 Suppl 110–16. 19393003 10.1111/j.1471-4159.2009.05939.x

[15] Hertz L, O'Dowd BS, Ng KT, Gibbs ME (2003) Reciprocal changes in forebrain contents of glycogen and of glutamate/glutamine during early memory consolidation in the day-old chick. Brain Res 994: 226–233. 14642648 10.1016/j.brainres.2003.09.044

[16] Simpson IA, Carruthers A, Vannucci SJ (2007) Supply and demand in cerebral energy metabolism: the role of nutrient transporters. J Cereb Blood Flow Metab 27: 1766–1791. 17579656 10.1038/sj.jcbfm.9600521 PMC2094104

[17] Mangia S, Simpson IA, Vannucci SJ, Carruthers A (2009) The in vivo neuron-to-astrocyte lactate shuttle in human brain: evidence from modeling of measured lactate levels during visual stimulation. J Neurochem 109 Suppl 155–62. 19393009 10.1111/j.1471-4159.2009.06003.x PMC2679179

[18] DiNuzzo M, Mangia S, Maraviglia B, Giove F (2010) Glycogenolysis in astrocytes supports blood-borne glucose channeling not glycogen-derived lactate shuttling to neurons: evidence from mathematical modeling. J Cereb Blood Flow Metab 30: 1895–1904. 20827264 10.1038/jcbfm.2010.151 PMC3002884

[19] DiNuzzo M, Mangia S, Maraviglia B, Giove F (2010) Changes in glucose uptake rather than lactate shuttle take center stage in subserving neuroenergetics: evidence from mathematical modeling. J Cereb Blood Flow Metab 30: 586–602. 19888285 10.1038/jcbfm.2009.232 PMC2949148

[20] Dinuzzo M, Mangia S, Maraviglia B, Giove F (2012) The role of astrocytic glycogen in supporting the energetics of neuronal activity. Neurochem Res 37: 2432–2438. 22614927 10.1007/s11064-012-0802-5 PMC4062197

[21] Xu J, Song D, Xue Z, Gu L, Hertz L, et al (2013) Requirement of glycogenolysis for uptake of increased extracellular K+ in astrocytes: potential implications for K+ homeostasis and glycogen usage in brain. Neurochem Res 38: 472–485. 23232850 10.1007/s11064-012-0938-3

[22] Govender D, Ramdial PK, Clarke B, Chetty R (2004) Clear cell (glycogen-rich) gastric adenocarcinoma. Ann Diagn Pathol 8: 69–73. 15060883 10.1053/j.anndiagpath.2004.01.002

[23] Brucklacher RM, Vannucci RC, Vannucci SJ (2002) Hypoxic preconditioning increases brain glycogen and delays energy depletion from hypoxia-ischemia in the immature rat. Dev Neurosci 24: 411–417. 12640180 10.1159/000069051

[24] Lau X, Zhang Y, Kelly DJ, Stapleton DI (2013) Attenuation of Armanni-Ebstein lesions in a rat model of diabetes by a new anti-fibrotic, anti-inflammatory agent, FT011. Diabetologia 56: 675–679. 23242170 10.1007/s00125-012-2805-9

[25] Vesce S, Rossi D, Brambilla L, Volterra A (2007) Glutamate release from astrocytes in physiological conditions and in neurodegenerative disorders characterized by neuroinflammation. Int Rev Neurobiol 82: 57–71. 17678955 10.1016/S0074-7742(07)82003-4

[26] Roulston CL, Callaway JK, Jarrott B, Woodman OL, Dusting GJ (2008) Using behaviour to predict stroke severity in conscious rats: post-stroke treatment with 3′, 4′-dihydroxyflavonol improves recovery. Eur J Pharmacol 584: 100–110. 18316070 10.1016/j.ejphar.2008.01.046

[27] Hossain MI, Roulston CL, Kamaruddin MA, Chu PW, Ng DC, et al (2013) A truncated fragment of Src protein kinase generated by calpain-mediated cleavage is a mediator of neuronal death in excitotoxicity. J Biol Chem 288: 9696–9709. 23400779 10.1074/jbc.M112.419713 PMC3617272

[28] Weston RM, Lin B, Dusting GJ, Roulston CL (2013) Targeting oxidative stress injury after ischemic stroke in conscious rats: limited benefits with apocynin highlight the need to incorporate long term recovery. Stroke Res Treat 2013: 648061. 23401848 10.1155/2013/648061 PMC3557625

[29] Singaravelu K, Lohr C, Deitmer JW (2006) Regulation of store-operated calcium entry by calcium-independent phospholipase A2 in rat cerebellar astrocytes. J Neurosci 26: 9579–9592. 16971542 10.1523/JNEUROSCI.2604-06.2006 PMC6674595

[30] Paquet M, Ribeiro FM, Guadagno J, Esseltine JL, Ferguson SS, et al (2013) Role of metabotropic glutamate receptor 5 signaling and homer in oxygen glucose deprivation-mediated astrocyte apoptosis. Mol Brain 6: 9. 23406666 10.1186/1756-6606-6-9 PMC3598502

[31] Thomas JA, Schlender KK, Larner J (1968) A rapid filter paper assay for UDPglucose-glycogen glucosyltransferase, including an improved biosynthesis of UDP-14C-glucose. Anal Biochem 25: 486–499. 5704765 10.1016/0003-2697(68)90127-9

[32] Zhang T, Wang S, Lin Y, Xu W, Ye D, et al (2012) Acetylation negatively regulates glycogen phosphorylase by recruiting protein phosphatase 1. Cell Metab 15: 75–87. 22225877 10.1016/j.cmet.2011.12.005 PMC3285296

[33] Walls AB, Sickmann HM, Brown A, Bouman SD, Ransom B, et al (2008) Characterization of 1,4-dideoxy-1,4-imino-d-arabinitol (DAB) as an inhibitor of brain glycogen shunt activity. J Neurochem 105: 1462–1470. 18221367 10.1111/j.1471-4159.2008.05250.x

[34] Shioda N, Han F, Moriguchi S, Fukunaga K (2007) Constitutively active calcineurin mediates delayed neuronal death through Fas-ligand expression via activation of NFAT and FKHR transcriptional activities in mouse brain ischemia. J Neurochem 102: 1506–1517. 17662023 10.1111/j.1471-4159.2007.04600.x

[35] Villar-Palasi C, Larner J (1970) Glycogen metabolism and glycolytic enzymes. Annu Rev Biochem 39: 639–672. 4320262 10.1146/annurev.bi.39.070170.003231

[36] DiNuzzo M, Mangia S, Maraviglia B, Giove F (2013) Regulatory mechanisms for glycogenolysis and K+ uptake in brain astrocytes. Neurochem Int 63: 458–464. 23968961 10.1016/j.neuint.2013.08.004 PMC4082998

[37] Cataldo AM, Broadwell RD (1986) Cytochemical identification of cerebral glycogen and glucose-6-phosphatase activity under normal and experimental conditions. II. Choroid plexus and ependymal epithelia, endothelia and pericytes. J Neurocytol 15: 511–524. 3018177 10.1007/BF01611733

[38] Lo EH, Dalkara T, Moskowitz MA (2003) Mechanisms, challenges and opportunities in stroke. Nat Rev Neurosci 4: 399–415. 12728267 10.1038/nrn1106

[39] Otori T, Friedland JC, Sinson G, McIntosh TK, Raghupathi R, et al (2004) Traumatic brain injury elevates glycogen and induces tolerance to ischemia in rat brain. J Neurotrauma 21: 707–718. 15253799 10.1089/0897715041269623

[40] Xu L, Sun H (2010) Pharmacological manipulation of brain glycogenolysis as a therapeutic approach to cerebral ischemia. Mini Rev Med Chem 10: 1188–1193. 20716050 10.2174/1389557511009011188

[41] Shearer J, Graham TE (2004) Novel aspects of skeletal muscle glycogen and its regulation during rest and exercise. Exerc Sport Sci Rev 32: 120–126. 15243208 10.1097/00003677-200407000-00008

[42] Choi HB, Gordon GR, Zhou N, Tai C, Rungta RL, et al (2012) Metabolic communication between astrocytes and neurons via bicarbonate-responsive soluble adenylyl cyclase. Neuron 75: 1094–1104. 22998876 10.1016/j.neuron.2012.08.032 PMC3630998

[43] Newgard CB, Hwang PK, Fletterick RJ (1989) The family of glycogen phosphorylases: structure and function. Crit Rev Biochem Mol Biol 24: 69–99. 2667896 10.3109/10409238909082552

[44] Lombardi MS, van den Tweel E, Kavelaars A, Groenendaal F, van Bel F, et al (2004) Hypoxia/ischemia modulates G protein-coupled receptor kinase 2 and beta-arrestin-1 levels in the neonatal rat brain. Stroke 35: 981–986. 15017017 10.1161/01.STR.0000121644.82596.7e

[45] Gurevich VV, Benovic JL (1993) Visual arrestin interaction with rhodopsin. Sequential multisite binding ensures strict selectivity toward light-activated phosphorylated rhodopsin. J Biol Chem 268: 11628–11638. 8505295

[46] Baillie GS, Sood A, McPhee I, Gall I, Perry SJ, et al (2003) beta-Arrestin-mediated PDE4 cAMP phosphodiesterase recruitment regulates beta-adrenoceptor switching from Gs to Gi. Proc Natl Acad Sci U S A 100: 940–945. 12552097 10.1073/pnas.262787199 PMC298705

[47] Premont RT, Inglese J, Lefkowitz RJ (1995) Protein kinases that phosphorylate activated G protein-coupled receptors. FASEB J 9: 175–182. 7781920 10.1096/fasebj.9.2.7781920

[48] Moore CA, Milano SK, Benovic JL (2007) Regulation of receptor trafficking by GRKs and arrestins. Annu Rev Physiol 69: 451–482. 17037978 10.1146/annurev.physiol.69.022405.154712

[49] Pierce KL, Lefkowitz RJ (2001) Classical and new roles of beta-arrestins in the regulation of G-protein-coupled receptors. Nat Rev Neurosci 2: 727–733. 11584310 10.1038/35094577

[50] Laureys G, Clinckers R, Gerlo S, Spooren A, Wilczak N, et al (2010) Astrocytic beta(2)-adrenergic receptors: from physiology to pathology. Prog Neurobiol 91: 189–199. 20138112 10.1016/j.pneurobio.2010.01.011

[51] Dong JH, Chen X, Cui M, Yu X, Pang Q, et al (2012) beta2-adrenergic receptor and astrocyte glucose metabolism. J Mol Neurosci 48: 456–463. 22399228 10.1007/s12031-012-9742-4

[52] Oz G, Kumar A, Rao JP, Kodl CT, Chow L, et al (2009) Human brain glycogen metabolism during and after hypoglycemia. Diabetes 58: 1978–1985. 19502412 10.2337/db09-0226 PMC2731528

[53] Mamedova LK, Shneyvays V, Katz A, Shainberg A (2003) Mechanism of glycogen supercompensation in rat skeletal muscle cultures. Mol Cell Biochem 250: 11–19. 12962138 10.1023/a:1024980710799

[54] Wender R, Brown AM, Fern R, Swanson RA, Farrell K, et al (2000) Astrocytic glycogen influences axon function and survival during glucose deprivation in central white matter. J Neurosci 20: 6804–6810. 10995824 10.1523/JNEUROSCI.20-18-06804.2000 PMC6772835

[55] Wang D, Luo P, Wang Y, Li W, Wang C, et al (2013) Glucagon-like peptide-1 protects against cardiac microvascular injury in diabetes via a cAMP/PKA/Rho-dependent mechanism. Diabetes 62: 1697–1708. 23364453 10.2337/db12-1025 PMC3636622

[56] Gold MG, Gonen T, Scott JD (2013) Local cAMP signaling in disease at a glance. J Cell Sci 126: 4537–4543. 24124191 10.1242/jcs.133751 PMC3795333

[57] Chen Y, Cann MJ, Litvin TN, Iourgenko V, Sinclair ML, et al (2000) Soluble adenylyl cyclase as an evolutionarily conserved bicarbonate sensor. Science 289: 625–628. 10915626 10.1126/science.289.5479.625

[58] Sorg O, Magistretti PJ (1992) Vasoactive intestinal peptide and noradrenaline exert long-term control on glycogen levels in astrocytes: blockade by protein synthesis inhibition. J Neurosci 12: 4923–4931. 1334506 10.1523/JNEUROSCI.12-12-04923.1992 PMC6575784

[59] Brown AM, Baltan Tekkok S, Ransom BR (2004) Energy transfer from astrocytes to axons: the role of CNS glycogen. Neurochem Int 45: 529–536. 15186919 10.1016/j.neuint.2003.11.005

[60] Stapleton D, Nelson C, Parsawar K, McClain D, Gilbert-Wilson R, et al (2010) Analysis of hepatic glycogen-associated proteins. Proteomics 10: 2320–2329. 20391537 10.1002/pmic.200900628 PMC2892038

[61] Parker GJ, Koay A, Gilbert-Wilson R, Waddington LJ, Stapleton D (2007) AMP-activated protein kinase does not associate with glycogen alpha-particles from rat liver. Biochem Biophys Res Commun 362: 811–815. 17767922 10.1016/j.bbrc.2007.08.080

