# Correction: Molecular Basis of Impaired Glycogen Metabolism during Ischemic Stroke and Hypoxia

**DOI:** 10.1371/journal.pone.0106173

**Published:** 2014-08-18

**Authors:** The PLOS ONE Staff

There is an error in affiliation 1 for authors Mohammed Iqbal Hossain and David Ian Stapleton. Affiliation 1 should be: Department of Physiology, The University of Melbourne, Melbourne, Victoria, Australia.
