# Supplementary material for: Molecular Basis of Impaired Glycogen Metabolism during Ischemic Stroke and Hypoxia
Source: PLoS One. 2014 May 23;9(5):e97570. doi: 10.1371/journal.pone.0097570 (PMC4032261; doi:10.1371/journal.pone.0097570)
Supplement: Supporting Information S1 — Supplement information and supporting figures. (DOC) [file pone.0097570.s001.doc]

Supplement Information

**Molecular basis of impaired glycogen metabolism during ischemic stroke and hypoxia**

Mohammed Iqbal Hossain,^1^ Carli Lorraine Roulston,^2^ and David Ian Stapleton^1^

^1^Department of Physiology and ^2^Department of Medicine, St. Vincent’s Campus, The University of Melbourne, Melbourne, Victoria, Australia.

**Protein kinase A (PKA) activity assay:**

PKA activity in brain lysates was determined using ProFluro PKA assay kit (Promega, WI, USA) according to manufacturer’s instruction. A standard kinase reaction with recombinant PKA (0.08 U) was performed in the Reaction Buffer with Bisamide Rhodamine 110 peptide substrate (PKA R110 Substrate). In this configuration, the PKA R110 Substrate is nonfluorescent . Following the kinase reaction, addition of a Termination Buffer, which contains a Protease Reagent, simultaneously stops the kinase reaction and removes amino acids specifically from the nonphosphorylated PKA R110 Substrate, resulting in the production of highly fluorescent Rhodamine 110. The phosphorylated PKA R110 Substrate is resistant to digestion by the Protease Reagent and remains nonfluorescent. Thus, the fluorescence intensity measured in the assay is inversely correlated with kinase activity.

Serial diluted PKA standards were assay according to the titration protocol. After titration, 25μl of whole brain lysate was assayed in triplicates in a 96-well microplate by adding 25μl of Kinase reaction buffer containing ATP. The reaction was stopped by adding 25 μl of protease solution and incubated for 30 min at room temperature. Finally a stabilizer solution was added and fluorescence was measured at an excitation wavelength of 485 nm and an emission wavelength of 530 nm in the Fluorskan Ascent fluorescence spectrophotometer (Thermo Electron corp.). Fluorescence results were then plotted into a standard PKA Fluorescence versus the Log10 PKA (units/well) curve. Curve fitting was performed using GraphPad Prism® 5.0 sigmoidal dose-response (variable slope). Standards PKA unit/well concentrations were converted to Log10. Samples fluorescence was then expressed as active PKA units by reconverting the Log10 of unit/well (associated to a corresponding fluorescence) to its antilog function. PKA Activity is expressed as the amount of active enzyme units per µg of protein.

**
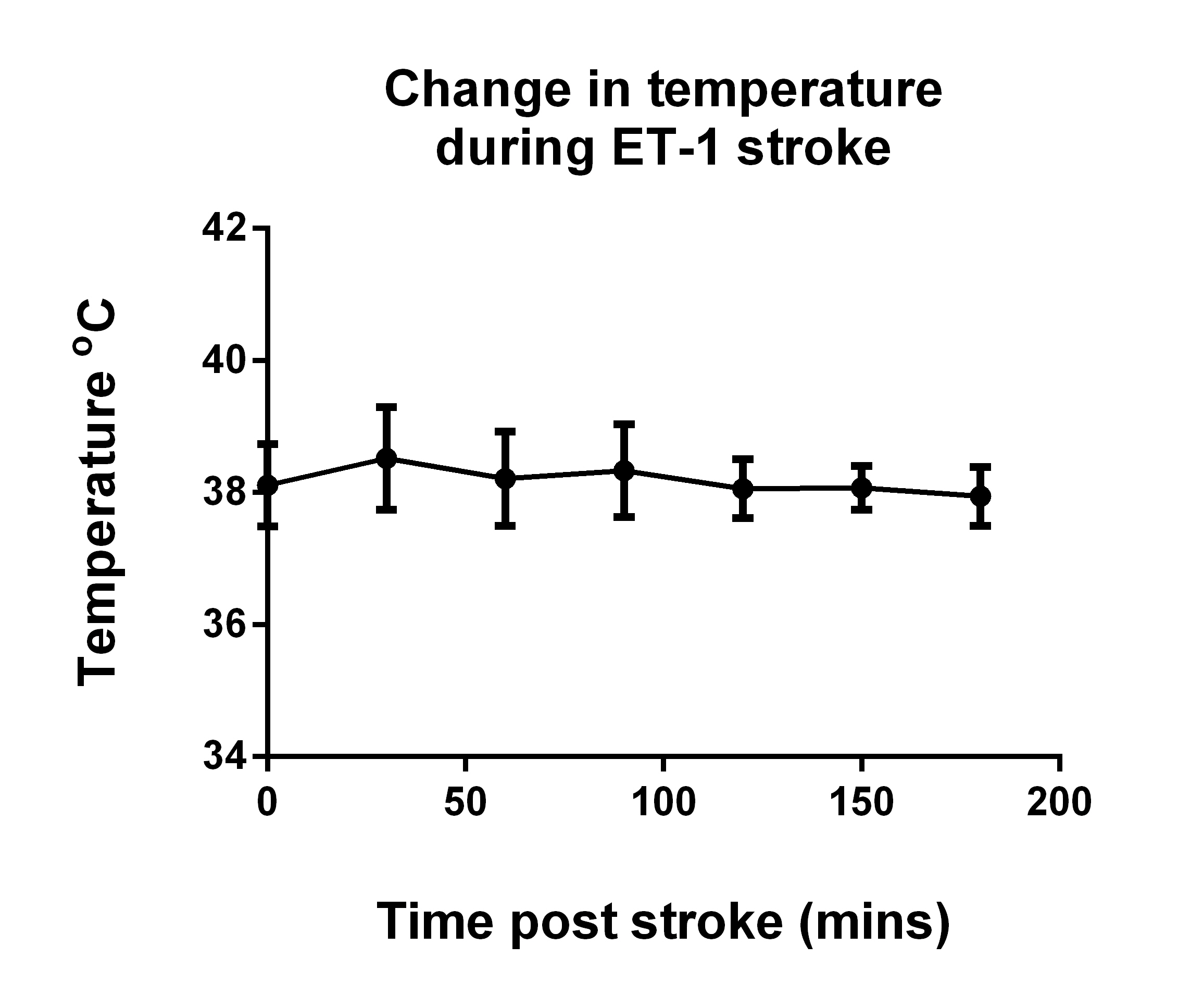
**

**Figure S1.** **Rectal temperatures in stroke-induced rats.** Representative figure showing the changes of rectal temperatures following ET-1 infusion commencing pre-stroke (0 mins) and then every 30 mins after stroke for 3 hours. Data are presented as mean ± SD (n = 12-15).

**
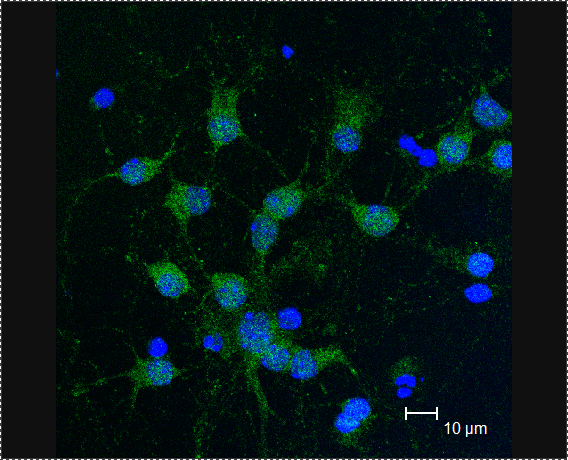
**

**Figure S2.** **Immunostaining of astrocytes with anti-GFPA antibody.** Representative figure of immunostaining with anti-GFPA antibody (green) of rat cerebellar astrocytes. Nucleus is stained with DAPI (blue). Cells were visualized and images were captured using Leica TCS SP2 confocal microscope with a 100 x 1.35 NA objective (Wetzler, Hassen, Germany).

**
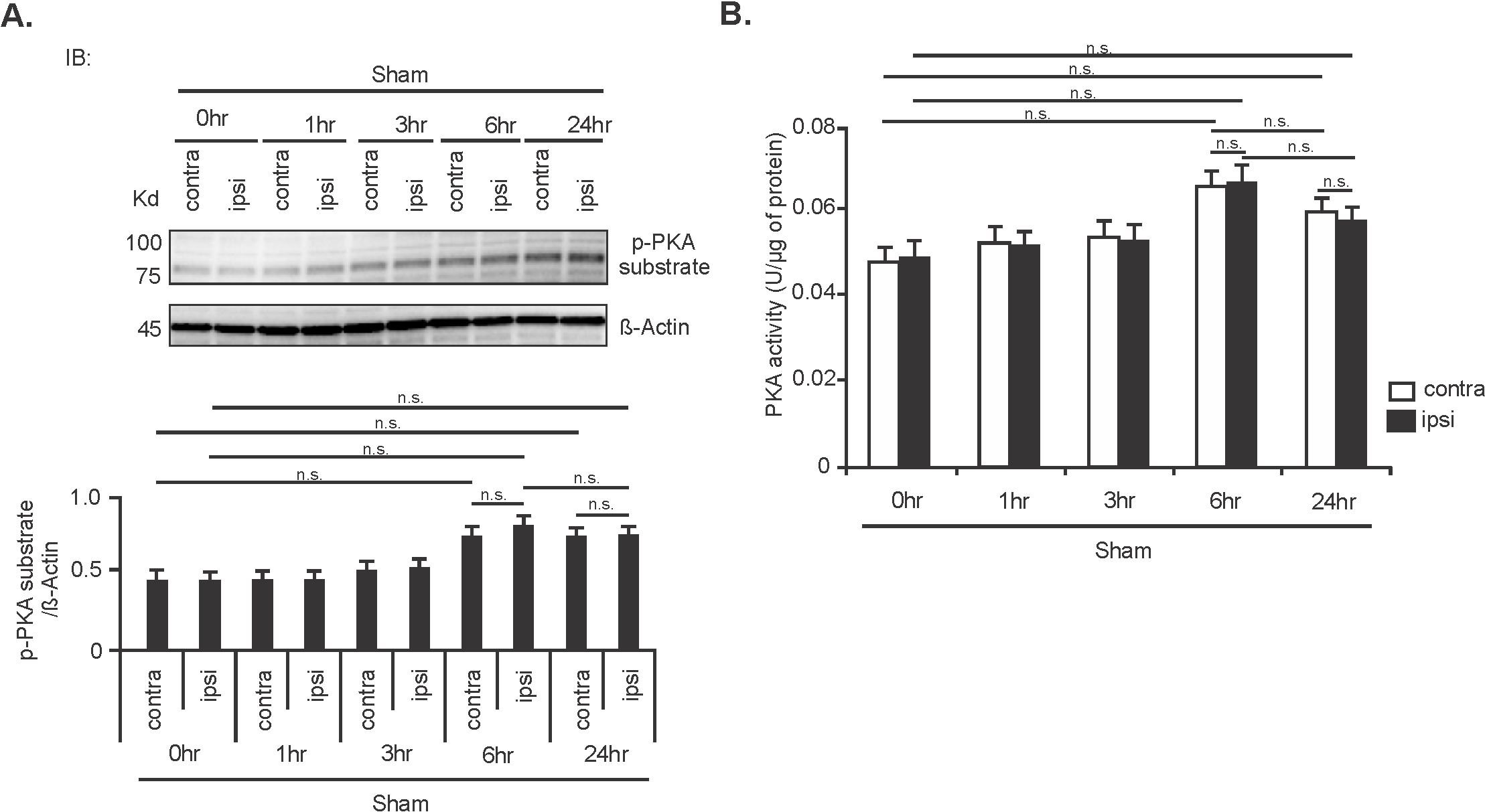
**

**Figure S3.** **PKA activity in sham rats. (A)** Representative immunoblot of p-PKA substrate of contralateral and ipsilateral hemispheres of sham rats after saline infusion at different time points. Phosphorylation level was normalized against β-Actin protein (mean ± SD, n = 5). (**B)** PKA activity in contralateral and ipsilateral hemispheres of sham rat brains (mean ± SD, n = 5 rats each group)

**
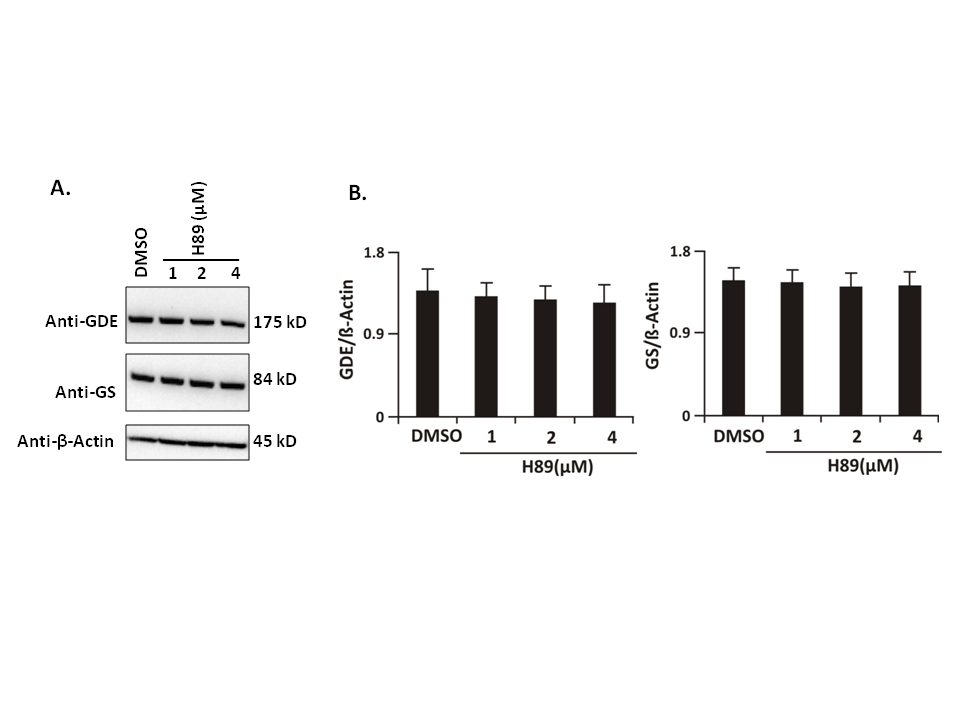
**

**Figure S4.** **Effect of PKA inhibitor on the expression levels of GDE and GS.**

**(A)** Expression levels of GDE and GS in primary cerebellar astrocytes treated with H89 for 24 hr. **(B)** Quantification of the expression level of GDE and GS in comparison to β-Actin protein. Data are expressed as mean ± SD, n=5.

**Reference:**

1. Leytus SP, Melhado LL, Mangel WF**: Rhodamine-based compounds as fluorogenic substrates for serine proteinas**es*. Biochem* J 1983**, 2**09(2):299-307.
